# Radiation Reduction Strategies for Endovascular Repair of Complex Aortic Aneurysms

**DOI:** 10.1016/j.ejvs.2025.12.025

**Published:** 2025-12-18

**Authors:** Yash K. Pandya, Muhammad S. Mazroua, Rudra V. Patel, Michel S. Makaroun, Nathan L. Liang

**Affiliations:** a University of Pittsburgh Medical Center, Division of Vascular Surgery, Pittsburgh, PA, USA; b University of Pittsburgh School of Medicine, Pittsburgh, PA, USA

**Keywords:** Digital zoom, Endovascular aortic repair, Fusion, Low dose fluoroscopy, Radiation reduction, Thoraco-abdominal aneurysm

The use of intensive radiation reduction strategies (RRS) for the endovascular repair of aortic pathology has been advocated in the literature, including in European Society for Vascular Surgery^1–3^ guidelines; however, reference level standards are lacking for complex aortic repair. This study hypothesised that the use of RRS would lower the radiation burden during endovascular repair of complex aortic pathology compared with standard radiation strategies (SRS).

A single centre, retrospective study was conducted comparing RRS with SRS (1 January 2018 – 31 December 2024). RRS incorporated a consistent five technique approach using two view onlay fusion, minimisation, or elimination of magnification in favour of digital zoom, dynamic collimation, limiting lateral views, and fluoroscopy loop use rather than digital subtraction angiography during contrast injections for non-critical imaging. SRS consisted of standard operator dependent procedures typically consisting of onlay fusion only. Procedures included fenestrated endovascular aortic repair (FEVAR) and branched repair with a thoraco-abdominal branch endoprosthesis (TAMBE; WL Gore, Flagstaff, AZ, USA). FEVAR procedures were stratified by a two vessel Zenith fenestrated device (ZFEN; Cook Medical, Bloomington, IN, USA), physician modified endovascular grafts with one to five fenestrations, and *in situ* laser fenestration. The outcomes of this study were cumulative dose (CD), dose area product (DAP), and dose per fluoroscopy minute (DPM). The study was exempt from informed consent by the University of Pittsburgh Human Research Protection Office (STUDY#20080199). Reporting followed Strengthening the Reporting of Observational Studies in Epidemiology (STROBE) guidelines.

The cohort consisted of 127 patients (71 FEVAR, 40 physician modified endovascular grafts, 11 TAMBE, and five *in situ* laser fenestration). SRS was used in 86 (67.7%) and RRS in 41 (32.3%). The RRS group (Table 1) had longer procedure and fluoroscopy times. CD did not differ between RRS and SRS in the overall cohort, but DAP and DPM were significantly lower in the RRS group. On multivariable linear regression, RRS was associated with a statistically significant reduction in DPM (*β* = −13.33; 95% confidence interval [CI] −21.64 – −5.02; *p* = .002), DAP (*β* = −134.26; 95% CI −247.36 – −21.16; *p* = .020), and CD (*β* = −1 182.04; 95% CI −1 924.47 – −439.61; *p* = .002) adjusting for age, sex, weight, and number of incorporated target vessels as a surrogate metric for anatomic and procedural complexity.

Two subgroup analyses were performed: one on the most recent 2024 subcohort to evaluate the learning curve and limit bias from improvement of the SRS group over time, and a second including only paravisceral and or thoraco-abdominal repairs to minimise selection bias from anatomic complexity. The 2024 subanalysis (*n* = 47) continued to demonstrate reduced DAP and DPM in the RRS cohort (Table 1), suggesting that the lower radiation doses were indeed associated with the RRS techniques rather than changes in practice. The high anatomic complexity subanalysis (SRS [*n* = 17], RRS [*n* = 39]) showed a statistically significant reduction for all median outcomes in the RRS group: CD (1 392 mGy [interquartile range {IQR} 845, 2 615] *vs.* 3 894 [IQR 2 708 – 5 250], *p* < .001); and DAP (125 Gy•cm^2^ [IQR 84 – 292] *vs.* 415 [IQR 304 – 620], *p* < .001).

This study demonstrated that the use of intensive RRS significantly decreases the radiation exposure to the patient and operating team. This adds to the existing literature^4–6^ and offers important data showcasing value for complex endovascular aortic repairs (EVARs). Hertault *et al.* examined differences in radiation exposure after the implementation of image fusion for standard and complex EVARs compared with a previously published cohort, showing a significant reduction in DAP.^5^ A meta-analysis of the utility of fusion showed a significant reduction in contrast volume use, fluoroscopy times, and procedure times for complex EVARs.^6^ In this study, despite increased complexity in the RRS group, doses decreased to below that of the less complex cases in the SRS group; the authors often performed complex multi-fenestrated and branched cases under 1 Gy.

This study had several limitations. RRS was used by one surgeon (N.L.L.), who performed the majority of the non-ZFEN complex aortic repairs as part of both the SRS and RRS groups. There was potentially unmeasured confounding due to this, including learning curves in both RRS and complex aneurysm repair, as well as the impact of a single surgeon (albeit high volume) championing awareness of these techniques. Independent audits to assess and standardize RRS techniques may be helpful for evaluation across multiple physicians. Additionally, this single site and imaging system may be difficult to generalise, given the specifics of any imaging setup. Also, due to the pragmatic real world nature of this study, there was inherent heterogeneity and selection bias within and between the cohorts that could not be completely accounted for even with subgrouping and multivariable analysis. Although the cohort consisted of a mix of native aneurysms and secondary procedures for type I and or III endoleaks, the effect of RRS on these salvage cases was not specifically explored in this study. Lastly, the study mainly focused on radiation exposure metrics reflecting dose to the patient and primary operator; individualised dedicated dosimetry is needed for future evaluation of staff dosing.

Given these study findings, centres performing EVARs in high volumes should consider structured implementation of intensive RRS whenever possible; indeed, the results from the RRS implementation have resulted in improved adoption of these techniques across the authors’ group practice. Future studies can focus on particular surgeon practices and behaviours in order to see if standardised practices can have an impact on radiation reduction across all centres.

## Figures and Tables

**Table 1.** Procedure and radiation outcomes compared by standard radiation strategies (SRS) *vs.* radiation reduction strategies (RRS) for the total cohort (*n*= 127) (2018 – 2024) and subcohort (2024 only).

Outcome	SRS total (*n* = 86)	RRS total (*n* = 41)	*p* value	SRS 2024 (*n* = 18)	RRS 2024 (*n* = 29)	*p* value
Fluoroscopy time – min	51 (35, 78)	61 (52, 96)	.012	51 (31, 70)	58 (46, 79)	.20
Dose area product – Gy·cm^2^	222 (139, 391)	125 (84, 275)	<.001	197 (135, 261)	105 (78, 143)	<.001
Cumulative dose – mGy	1 500 (1 155, 3 135)	1 291 (820, 2 611)	.12	1 430 (1 153, 2 433)	975 (733, 1 517)	.14
Cumulative dose/minute – mGy/min	34 (21, 49)	21 (14, 30)	<.001	26 (21, 42)	19 (14, 26)	.019

Data are presented as *n* (median interquartile range). SRS = standard radiation strategies; RRS = radiation reduction strategies.
